# Preferred Orientation Contribution to the Anisotropic Normal State Resistivity in Superconducting Melt-Cast Processed Bi_2_Sr_2_CaCu_2_O_8+δ_

**DOI:** 10.3390/ma10050534

**Published:** 2017-05-15

**Authors:** Aline Dellicour, Benedicte Vertruyen, Mark O. Rikel, Luca Lutterotti, Alain Pautrat, Bachir Ouladdiaf, Daniel Chateigner

**Affiliations:** 1 GREENMAT, CESAM Research Unit, Institute of Chemistry, University of Liege, Sart-Tilman, 4000 Liege, Belgium; a.dellicour@ulg.ac.be (A.D.); b.vertruyen@ulg.ac.be (B.V.); 2 Normandie Université, CRISMAT-ENSICAEN-UCN UMR 6508 CNRS, 6 Bd. Maréchal Juin, 14050 Caen, France; alain.pautrat@ensicaen.fr; 3 Nexans Superconductors, 30179 Hannover, Germany; rikel@d-nano.com; 4 Deptment of Industrial Engineering, University of Trento, via Sommarive, 9–38123 Trento, Italy; luca.lutterotti@unitn.it; 5 Institut-Laue-Langevin, Bd. Jules Horowitz, 38042 Grenoble, France; ouladdiaf@ill.eu

**Keywords:** texture, neutron diffraction, superconductor, Bi_2_Sr_2_CaCuO_8_, anisotropy, resistivity

## Abstract

We describe how the contribution of crystallographic texture to the anisotropy of the resistivity of polycrystalline samples can be estimated by averaging over crystallographic orientations through a geometric mean approach. The calculation takes into account the orientation distribution refined from neutron diffraction data and literature values for the single crystal resistivity tensor. The example discussed here is a melt-cast processed Bi_2_Sr_2_CaCu_2_O_8+δ_ (Bi-2212) polycrystalline tube in which the main texture component is a <010> fiber texture with relatively low texture strength. Experimentally-measured resistivities along the longitudinal, radial, and tangential directions of the Bi-2212 tube were compared to calculated values and found to be of the same order of magnitude. Calculations for this example and additional simulations for various texture strengths and single crystal resistivity anisotropies confirm that in the case of highly anisotropic phases such as Bi-2212, even low texture strengths have a significant effect on the anisotropy of the resistivity in polycrystalline samples.

## 1. Introduction

The Bi_2_Sr_2_CaCu_2_O_8+δ_ (Bi-2212) high-temperature superconductor is a typical example of a compound characterized by a large electro-magnetic anisotropy, resulting from a strong crystallographic two-dimensionality (*ab* plane vs. *c*-axis). In single crystals above the critical temperature (T_C_), Bi-2212 displays an electrical resistivity anisotropy ratio ρ_c_/ρ_ab_ up to ~10^4^–10^5^ [1,2]. In the superconducting state, it is known that grain misorientation strongly suppresses the critical current density J_c_, as also observed in YBa_2_Cu_3_O_7-x_ [3]. For example, in Bi-2212 films, Mori et al. [4] reported a difference of two orders of magnitude between intra-grain J_c_ (10^5^ A/cm^2^) and inter-grain J_c_ (10^3^ A/cm^2^) for a 24° tilt angle between two grains—much larger values than for YBa_2_Cu_3_O_7_ [5]. In principle, the higher J_c_ sensitivity to grain misorientation can be expected because of the larger electronic anisotropy of Bi-2212 [6].

Despite this sensitivity, Bi-2212 polycrystalline samples containing high-angle grain boundaries can exhibit unexpectedly large macroscopic J_c_-values—a still-unexplained behavior, particularly in weakly-textured samples. The so-called melt-cast processed (MCP) Bi-2212 bulk exhibit intriguingly large J_c_(77K) up to 5 kA/cm^2^, while their crystallographic texture strength is weak [7]. Note that these J_c_(77K, self-field) values are almost the same or even larger than J_c_ values reported for highly-textured bulk Bi-2212 samples produced by hot forging [8] or partially melt processed in magnetic field [9].

In order to provide some insight into this apparently strange behavior, we studied the effect of generally very weak texture on the properties of MCP bulk Bi-2212. The present work reports our results on normal state resistivity anisotropy. In particular, we studied the contribution of texture to the anisotropy of the normal state resistivity in such samples. Using (i) the experimentally determined orientation distribution (OD) of crystallites and (ii) literature data for the single crystal resistivity tensor, the macroscopic normal state electrical resistivities along the three tube directions (longitudinal, radial, and tangential) were simulated through OD-weighted geometric mean tensor calculations (see next section). The simulated values were then compared to experimental measurements in order to discuss to what extent the weak texture strengths are able to generate anisotropy in the macroscopic properties. We found that the calculations predict the anisotropy of resistivity rather well, but give significantly higher absolute values, which we consider as evidence that the normal current is mostly confined in the *ab*-planes with very small (if any) *c*-axis contribution. This is also supported by the normalized temperature dependence of resistivity that (contrary to other bulk Bi-2212) coincides with the normalized in-plane resistivity of single crystals.

## 2. Materials and Methods 

### 2.1. OD-Weighted Resistivity Simulations

For perfectly randomly oriented samples with theoretical density, the simplest calculation of a macroscopic property T^M^ corresponds to the arithmetic mean <T> of the single crystal property tensor T. However, in the case of properties such as resistivity ρ and its inverse, conductivity σ, this procedure does not ensure that ρ^M^ = 1/σ^M^, because <ρ> is not necessarily equal to <σ>^−1^ = <ρ^−1^>^−1^. This issue was extensively discussed in the literature for the case of another pair of mutually inverse properties (e.g., elastic stiffness and compliance) [10,11], where stress and strain homogeneity cannot be simultaneously respected with an arithmetic mean procedure. Matthies and Humbert [12] showed that in this case geometric mean averaging resulted in homogeneous properties [T] = [T^−1^]^−1^, and that this procedure yielded values in good agreement with more complex visco-plastic self-consistent approaches—even for textured polycrystals.

Similarly, the geometric mean averaging method will be applied here in order to ensure the macroscopic homogeneity for resistivity and conductivity:

ρ^M^ = [ρ] = [ρ^−1^]^−1^ = (σ^M^)^−1^, (1)

The calculation (see Appendix A for equations) must take into account (i) the non-random texture of the sample, represented by the orientation distribution OD; and (ii) the anisotropy of the resistivity, requiring the description of the single crystal resistivity as a second rank tensor. The output of the calculation will be a set of three values, corresponding to the calculated resistivity along the X, Y, and Z tube directions (denoted hereafter as ρ_X_^calc^, ρ_Y_^calc^, ρ_Z_^calc^).

### 2.2. Experimental

We studied a 350 mm-long melt-cast-processed Bi-2212 tube with inner and outer diameters of 35 mm and 50 mm, respectively. This tube was taken from Nexans Superconductors GmbH production for Bi-2212-based fault current limiters being qualified to have self-field J_c_(77, sf) = 600 A/cm^2^ averaged over the whole (7.5 mm) thickness [13]. A similar tube was previously characterized for its ability to be used as magnetic shield [14]. The tube was produced from a mixture of oxides in molar ratios Bi:Sr:Ca:Cu 2.00(3):1.99(3):0.87(3):2.04(3) with admixture of 0.10(2) BaO and 0.41(3) SrSO_4_ by melting at 1100 ± 20 °C in Pt crucibles and casting in rotating moulds. The melt-cast tube was heat-treated to form Bi-2212 phase with the final annealing at 840 °C in air to tune nominal O index δ = 0.200 ± 0.005 (according to [15]), optimized for applications at 77 K [7,13]. Cooling down in air after annealing resulted in some over-doping (Δδ ~ 0.02) in the approximately 0.5 mm-thick layers at the outer and inner surfaces of the tube.

**Figure 1:**
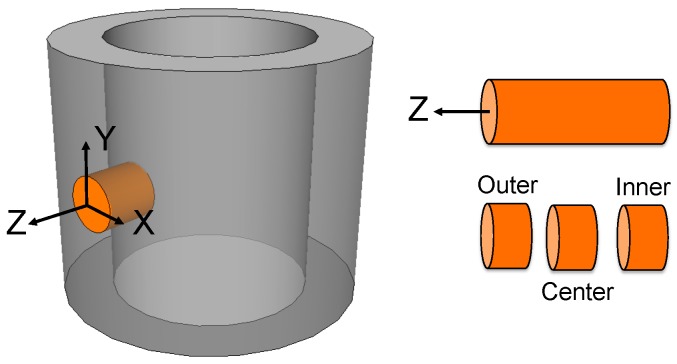
Reference frame of the Bi-2212 tube and sample cutting procedure.

**Figure 2:**
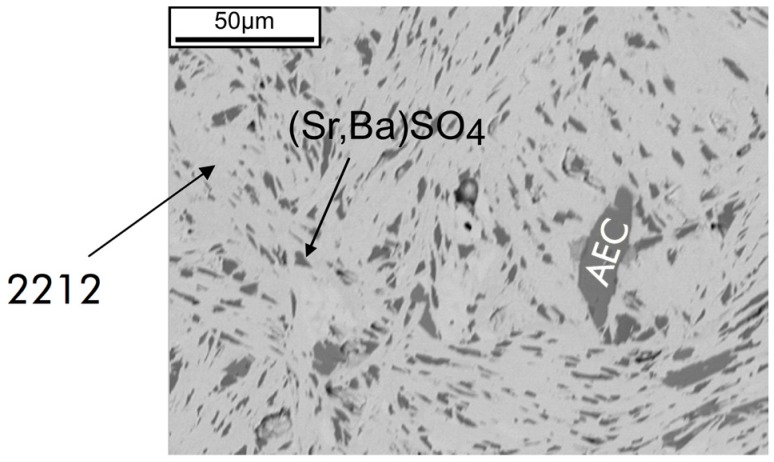
SEM (backscattered electron, BSE) image of a polished cross-section in the central part of the tube showing major second phases (Ba_0.13_Sr_0.87_)SO_4_ and (Sr_0.6_Ca_0.4_)CuO_2_ (1:1AEC) in the Bi-2212 matrix.

For texture measurements, the tube reference frame was defined as shown in Figure 1, with X tangent to the tube surface, Y along the tube axis and Z in the radial direction. A 10-mm-diameter, 7-mm-long cylinder was drilled along the tube radial Z direction and cut in three 2-mm thick samples. The central part of the tube wall is known to be fully dense with rather uniform microstructure [7] (see also Figure 2). The texture of Bi-2212 in that sample was studied using neutron diffraction at the D1B-ILL beamline (λ = 2.5249 Å). Diffraction patterns were recorded with a 5° grid resolution in tilt (χ) and azimuth (φ) angles at an incident ω angle of 44.95° [16]. The sample was oriented with Z// φ axis and Y// χ axis (at φ = 0). All diffraction data were analyzed within the combined analysis frame [16] using the MAUD software [17]; texture calculation was carried out with the extended-Williams-Imhof-Matthies-Vinel (E-WIMV) model. The OD-weighted geometric mean tensor calculations for simulation of the macroscopic resistivity values were also carried out using the MAUD Software. Neutron diffraction experiments were performed on five samples extracted from different positions in the tube. Data were recorded and analyzed according to the above-described procedure. We observed roughly 10% variation in OD-weighted calculated resistivities.

Electrical resistivity along the X, Y, or Z tube directions (noted hereafter ρ_X_^exp^, ρ_Y_^exp^, ρ_Z_^exp^) was measured using a PPMS 14T system (Quantum Design, San Diego, CA, USA). Bars were cut from the central part of the tube (from z ~ 1.5 to 5.5 mm; see Figure 1), with their longest dimension parallel to X, Y, or Z; sample dimensions were 1 × 1 × 7 mm^3^ (measurement in X and Y directions) or 1 × 1 × 4 mm^3^ (measurement in Z direction). Indium contacts were attached to the 1 × 1 mm^2^ faces, and resistivity was measured by the standard four-probe technique with a 5 mA current. The sample-to-sample reproducibility in room-temperature resistivity was better than 5%.

## 3. Results

### 3.1. Microstructure and Texture Characteristics

Preliminary characterization by electron microscopy and energy dispersive X-ray analysis revealed Bi_2.15(2)_Sr_2.08(2)_Ca_0.81(2)_Cu_1.96(3)_O_8+δ_ as the main phase (85 ± 5 vol. %), together with (Ba_0.13_Sr_0.87_)SO_4_ (10 ± 2 vol. %) and (Sr_0.6_Ca_0.4_)CuO_2_ (1:1AEC, Alkaline Earth Cuprate) as major second phases (Figure 2).

In view of the analysis of the neutron data for texture characterization, starting values for the cell parameters of the three phases were obtained by Rietveld refinement of a Cu K_α1_ X-ray diffraction pattern collected on a powder prepared by grinding a piece of the tube from the central part. Atomic positions were taken from CIF files 1000285 (Bi-2212) and 9009506 [(Sr,Ba)SO_4_] of the Crystallographic Open Database (COD) [18] and from PDF 04-007-4981 (1-1AEC) of the PDF-4+ reference database.

**Figure 3:**
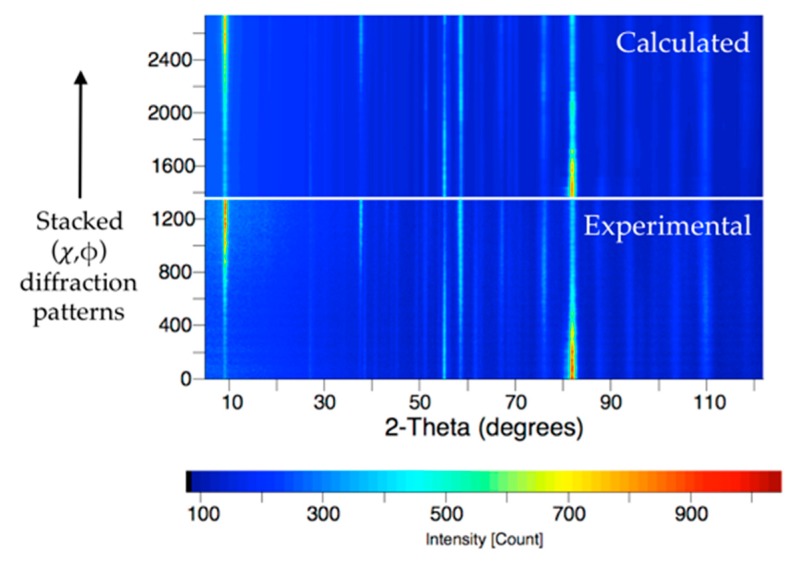
Measured (**bottom**) and calculated (**top**) diffraction patterns showing the good refinement quality using combined analysis [16]. All (χ,φ) diffraction patterns are stacked along the vertical axis, with intensity given as a color code according to the bottom bar.

Figure 3 compares the measured D1B data (patterns 0 to 1368, bottom of the graph) with the calculated patterns (1369 to 2736, top of the graph) obtained using the E-WIMV algorithm of the MAUD software and refining 2θ shift, cell parameters, scale factors, instrumental, and background functions. Simple visual inspection of this figure shows that the refinement successfully reproduces the variations in intensities of the reflections. The refinement converges with a satisfactory goodness of fit (GOF) of 1.47. A refinement using a tetragonal instead of an orthorhombic cell for Bi-2212 resulted in a much-degraded fit (GOF = 2.8).

**Figure 4:**
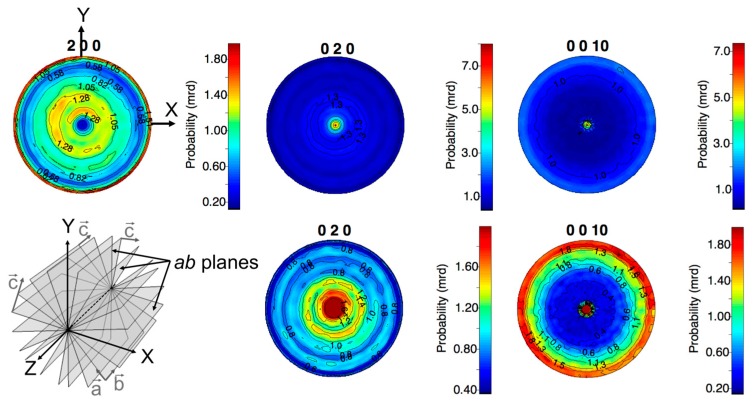
Bi-2212 {200}, {020}, and {0010} normalized pole figures refined from D1B data. The {020} and {0010} are also shown with a different color scale to provide more detail in the <2 multiples of a random distribution (m.r.d.) range. The main texture component is schematized in the bottom-left panel.

The {200}, {020}, and {0010} normalized pole figures refined for the orthorhombic Bi-2212 phase are shown in Figure 4. The Bi-2212 texture is weak, characterized by maxima of orientation distribution densities not larger than seven multiples of a random distribution (m.r.d.). The {200} and {0010} pole figures show equator reinforcements; i.e., *a*- and *c*-axes are preferentially oriented in the XOY sample plane. This is a <010>-fiber texture, coherent with the reinforcement at the center of the {020} pole figure. As schematized in the bottom-left panel of Figure 4, this texture component corresponds to *ab*-planes parallel to and randomly distributed around the Z-axis of the tube. The reinforcement of the center of the {0010} pole figure indicates presence of a minor texture component (less than 10 vol. %), with the *c*-axis oriented along the Z tube direction.

The objective is to use these texture results to discuss the contribution of the orientation distribution to the anisotropy of electrical resistivity along the X,Y,Z tube directions.

### 3.2. Electrical Resistivity

**Figure 5:**
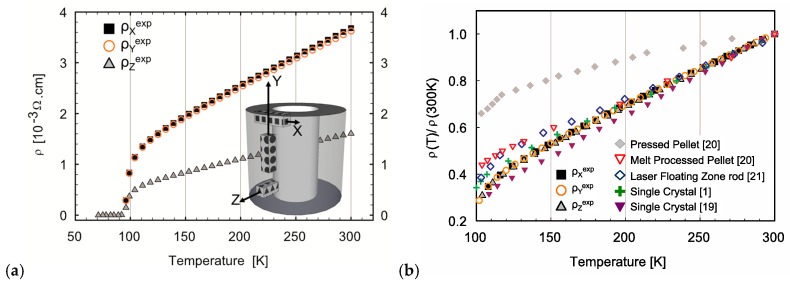
(**a**) Temperature dependence of the resistivity measured on bar specimens cut along the X, Y, and Z directions of the tube (geometry in inset); (**b**) its comparison with the literature data for single crystals [1,19] and Bi-2212 bulk samples processed using other techniques [20,21].

Figure 5a displays the measured resistivity vs. temperature curves of bars cut with their long axis parallel to the X, Y, or Z direction of the tube. All samples exhibit metallic behavior above the superconducting transition at T_c_ = 92 K. The normal state resistivity curves along the X and Y directions coincide within the error bar, while the resistivity along the Z direction is markedly smaller. The measured anisotropic resistivity ratio ρ_Y_/ρ_Z_ is around 2.3, roughly constant in the 100–300 K range, with a slight increase near the superconducting transition. Note that the normalized resistivity ρ(T)/ρ(300 K) (Figure 5b) is the same for all three directions, and almost coincides with the literature data on the in-plane resistivity ρ_ab_(T)/ρ_ab_(300 K) in single crystals—a behavior different from that in Bi-2212 bulk samples processed using other techniques.

### 3.3. Modeling the Resistivity 

In the case of a crystal with orthorhombic point group symmetry, such as Bi-2212, the resistivity tensor of the single crystal has only three independent resistivity components: ρ_11_, ρ_22_, and ρ_33_. The single crystal resistivity data ρ_ab_(T) and ρ_c_(T) were taken from the work of Watanabe et al. [1], who showed that changes of O contents δ from strongly overdoped to strongly underdoped states lead to an increase of the room-temperature resistivities from 0.2 to 0.9 mOhm·cm (ρ_ab_) and from 2 to 8 Ohm·cm (ρ_c_). The absolute values of δ were not measured in that work, but only assigned to vary from 0.21 to ≈0.27 based on a comparison of annealing conditions of studied single crystals with those published in the literature for polycrystalline samples [22]. Note that the determination of the absolute values of δ in Bi-2212 can be done only with rather large errors (σ_δ_ = ±0.02) [15,23]. The absolute scales reported in the literature significantly differ, but give essentially the same (within ±0.002 precision) scales relative to annealing conditions.

Comparison based on the annealing conditions shows that the absolute scales of O index δ according to Schweizer et al. [15] used in this and our previous studies and by Watanabe et al. [1] are shifted by Δδ = 0.040 ± 0.005, so that the nominal δ ≈ 0.202 in our notation corresponds to δ* ≈ 0.245 in the notations of Watanabe et al. [1]). We took the data ρ_ab_(300 K) = 0.43 mΩ·cm and ρ_c_(300 K) = 4000 mΩ·cm reported by Watanabe for δ* = 0.245 as single crystal values. This choice may introduce some errors that we discuss below.

**Figure 6:**
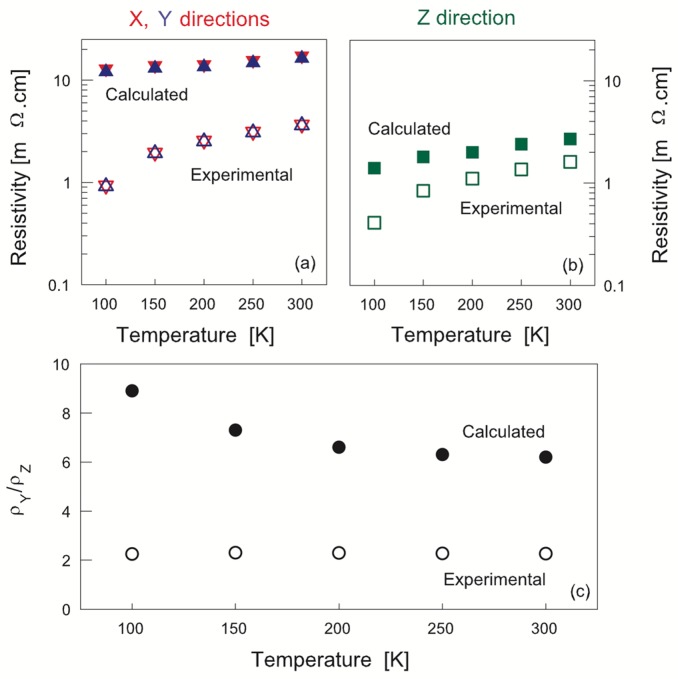
(**a**,**b**) Comparison of experimental resistivity values (open symbols) with values calculated by averaging over crystallite orientations (full symbols) using the orientation distribution refined from the measured pole figures with the extended-Williams-Imhof-Matthies-Vinel (E-WIMV) iterative algorithm; (**c**) Calculated (full symbols) and experimental (open symbols) resistivity ratios.

The resistivity values calculated by weighing over crystallite orientations from the refined orientation distribution (OD) determined from neutron pole figures and the measured resistivities are compared in Figure 6a,b. The calculated OD-weighted resistivities are ρ_X_^calc^ ≈ ρ_Y_^calc^ ≈ 10 mΩ·cm (Figure 6a) and ρ_Z_^calc^ ≈ 3 mΩ·cm (Figure 6b). As expected, the calculation reproduces the lower resistivities in the Z direction compared to X and Y. However, the OD-weighted calculated values are 1.5 times (Z direction) to 10 times (X and Y directions) larger than the experimental ones, depending on temperature.

As it follows from Figure 6c, the experimental resistivity ratio (ρ_Y_/ρ_Z_ = 2.3) is smaller than the one obtained from the OD-weighted calculated resistivities (7 to 9) and is not temperature-dependent, while the calculated resistivity ratio is temperature-dependent. It is thus instructive to assess to what extent this discrepancy may originate from the changes in parameters of our model.

**Figure 7:**
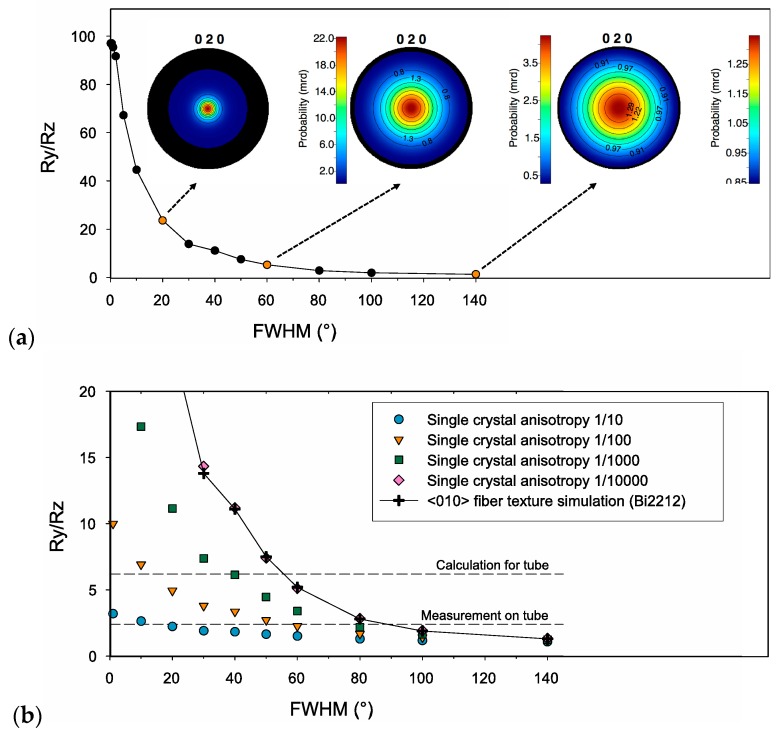
The anisotropic resistivity ratio of the Bi-2212 phase for (**a**) a simulated fiber texture characterized by a ρ_33_/ρ_11_ = 10^4^ anisotropy with varying Gaussian full-widths and (**b**) simulated fiber textures with varying anisotropy and Gaussian full-widths and their comparison to melt-cast tube. FWHM: full width at half maximum.

In melt-cast-processed samples, the orientation densities do not typically exceed a few m.r.d., or are even close to randomly-oriented samples. However, the large intrinsic anisotropy of Bi-2212 (10^4^ or more) compensates for the low texture strength. As a further illustration of this combination between texture strength and resistive tensor anisotropy, simulations were performed for a <010>-fiber texture component (i.e., with *b* // Z) with decreasing maximum orientation densities; i.e., increasing full widths at half maximum (FWHMs) of the Gaussian distribution of the fiber component in the orientation space. The anisotropic resistivity ratio ρ_Y_^calc^/ρ_Z_^calc^ was then calculated for each texture simulation and for several anisotropies of the single crystal. Figure 7a plots the results of simulations for the Bi-2212 phase, characterized by a very high anisotropy (i.e., resistivity tensor components ρ_33_/ρ_11(=_ρ_22)_ = 10^4^). It is worth noting that the ρ_Y_^calc^/ρ_Z_^calc^ ratio for a “perfect” <010>-fiber texture is only about 10^2^: this highlights the difference between the melt-cast-processed tube studied here and samples prepared by technologies favoring <001>-fiber texture (tapes or uniaxially-pressed bulks, for example). In the present case of <010>-fiber texture, the orientation of *ab*-planes (see Figure 4) is such that current flow is possible in all directions. Regarding the influence of texture strength, Figure 7a shows that the ρ_Y_^calc^/ρ_Z_^calc^ ratio decreases from ~100 to ~45 for a 10° FWHM of dispersion and to ~10 for a 40° FWHM. Larger dispersions up to 140° reduce the resistivity ratio to ~2, but for these smaller texture strengths, the dependence of the anisotropy on the FWHM becomes less marked: at low orientation levels, further randomization of conducting planes does not significantly modify the carrier paths. ρ_Y_^calc^/ρ_Z_^calc^ ratios of 10 or less may seem modest, but additional simulations carried out for less anisotropic phases (ρ_33_/ρ_11_ of 10^3^, 10^2^ or 10) show (Figure 7b) that a factor of ~7 such as that calculated for the melt-cast-processed tube would require a much stronger texture if ρ_33_/ρ_11_ = 10^3^ or 10^2^, or cannot be achieved if ρ_33_/ρ_11_ = 10. Consequently, thanks to the very high resistive anisotropy of Bi-2212 single crystals, melt-cast-processed bulks are still exhibiting comparatively strong anisotropies of their resistivity.

## 4. Discussion

### 4.1. Texture Development in MCP Bi-2212

The preferred orientation in the central part of MCP Bi-2212 tubes with the *ab*-planes (and hence the CuO_2_-planes) parallel to the radial (Z) tube direction was discussed earlier as a possible reason for the anisotropy of magnetic properties [14] and intensity redistribution in XRD patterns [15]. Our results (Figure 4) fully confirm this qualitative picture, showing the dominance of fiber texture component with *c* axis perpendicular to the radial direction.

The origin of this texture component is likely related to thermal gradients along the radial direction that determine the growth direction of (Sr,Ba)SO_4_ dendrites, (1:1) AEC and Bi-2201 phases precipitating at different temperatures during cooling of the melt, which finally solidifies as a 0.32Cu_2_O + 0.68Bi_2.2(1)_Sr_2.1(1)_Ca_0.7(1)_O_x_ eutectic [15]. During postannealing of the melt-cast material at temperatures higher than the eutectic temperature (741 ± 2 °C [15]), these precipitates may act as template phases for growing platelets of Bi-2212. The gradient of oxygen concentration whose diffusion is necessary for Bi-2212 formation is another reason for the radial direction Z being the direction of Bi-2212 platelet growth.

The observed strong difference in 200 and 020 pole figures (Figure 2) may reflect the anisotropy of in-plane growth of Bi-2212, for which the growth rate in the modulation-free direction is known to be an order of magnitude faster than in the direction of modulation (e.g., [24,25]). The structural model of Bi-2212 that takes into account modulation in one of in-plane directions should be used to check this hypothesis.

### 4.2. Resistivity and Its Anisotropy-Possible Reasons for Discrepancy between the Experiment and Model Calculations

Qualitatively, the fact that the measured resistivity is lowest along the Z direction is coherent with the main texture component; i.e., the preferential orientation of the *ab*-planes (and hence the CuO_2_-planes) parallel to the Z tube direction. In order to go beyond this qualitative observation, we used the refined crystallite orientation distribution and measured single-crystal resistivity data to model the resistivity anisotropy of the sample and compared it to the measured one.

Such a comparison shows significant discrepancies between the experimental data and modeling results. The simulation gives OD-weighted calculated macroscopic room-temperature resistivity values from 1.5 (Z direction, Figure 6b) to 10 times (X and Y directions, Figure 6a) larger than the experimental ones. The difference between experiment and calculations essentially increases at low temperatures (Figure 6c). These discrepancies require discussion.

Simulations done to assess the accuracy of our calculations (see Figure 7 and its discussion) show that the experimental uncertainties in OD function result in much smaller errors than the observed discrepancies, so either the input parameters of our model or some model assumptions appear to be incorrect.

Several potential reasons for the difference between the measured and calculated resistivities can be considered. First, our samples have the cation composition (Sr/Ca ≈ 2.60) that significantly differs from that of single crystals studied by Watanabe et al. [1] (Sr/Ca ≈ 2.0–2.2), and processing under similar conditions may result in different O contents δ and different carrier doping levels [26]. Unfortunately, to our knowledge, no data on the resistivity of single crystals having different cation compositions are available in the literature. However, we do not anticipate that the difference in ρ_ab_ due to the difference in cation compositions may explain the observed discrepancies between experimental and calculated ρ_X_, ρ_Y_, and ρ_Z_. Even larger differences in cation composition (Sr/Ca = 1.35 to 2.6) were found to have only minor (<5%) effects on the room-temperature resistivity of MCP bulk samples prepared using the same pO_2_-T trajectories [27].

The in-plane anisotropy of resistivity that is not taken into account in our model calculations can be another reason for the observed discrepancies. Makise et al. [28] report the ρ*_b_*/ρ*_a_* ratio (*b* is the modulation direction) varying from 1.5 for single crystals with Sr/Ca ≈ 1.6 to ~3 for Sr/Ca ≈ 2.1. However, this result should be double-checked, because such a strong anisotropy must lead to at least 50% sample-to-sample irreproducibility in ρ_ab_ values, which was not reported in other studies of resistivity in Bi-2212 single crystals. Even in the case of large ρ*_b_*/ρ*_a_* ≈ 3, due to rather weak texture and the random distribution of *ab*-planes around the Z direction of the tube sample, OD-weighted calculated resistivities are on the same order of magnitude as the ones calculated with ρ*_b_* = ρ*_a_*. OD-weighted calculated resistivities at 300 K are, respectively, ρ_X_^calc^ = 21 mΩ·cm, ρ_Y_^calc^ = 23 mΩ·cm, and ρ_Z_^calc^ = 4.5 mΩ·cm when calculated with ρ_b_/ρ_a_ ≈ 3 and ρ_X_^calc^ = 17 mΩ·cm, ρ_Y_^calc^ = 16 mΩ·cm, and ρ_Z_^calc^ = 2.7 mΩ·cm when calculated with ρ*_b_* = ρ*_a_*.

The essentially effective-medium model that we have used for resistivity calculations (see Appendix A) does not consider short-range correlations in grain alignment—in other words, the special character of grain boundaries that may lead to the formation of rather long percolative current paths, which due to high anisotropy are nevertheless more conductive than predicted by the effective medium calculations. Our data on the normalized temperature dependence of resistivity in Figure 5b support this idea. The normalized resistivity in our samples is within the error (independent of the cutting direction), and coincides with the normalized in-plane resistivity ρ*_ab_*(*T*) for single crystals, which is not the case for Bi-2212 bulk samples processed using other techniques (not MCP). In the latter samples, a significant enhancement of the resistivity ratio R(T)/R(300 K) is observed below 200 °C, likely due to the *c*-axis constituent of the current path. The absence of such an enhancement in the MCP bulk samples may indicate that the current path is almost fully confined to the *ab*-planes. The special structure of high-angle grain boundaries in MCP bulk Bi-2212 [29,30] could be the reason for such behavior.

The *c*-axis resistivity is known to have a semiconductor-like behavior, with ρ_c_(100 K)/ρ_c_(RT) ~ 3 [1]. As far as the effective medium model takes the *c*-axis contribution into account (and due to texture, this contribution is stronger for y and z directions), the calculated ratio ρ_Y_/ρ_Z_ is temperature-dependent. On the other hand, Figure 5 shows that experimental ρ_X_, ρ_Y_, and ρ_Z_ all have exactly the same normalized temperature dependence, which makes the experimental anisotropy ratio ρ_Y_/ρ_Z_ independent of temperature. This difference is thus a strong argument in favor of the hypothesis that *c*-axis contribution in the normal conduction path is negligible in our samples.

## 5. Conclusions

The example of the melt-cast-processed (MCP) Bi-2212 tube studied here illustrates that quantitative texture analysis provides the way for the calculation of orientation-distribution-weighed resistivities through tensor homogenization using geometric mean averaging. The calculated values obtained using the orientation distribution refined from neutron diffraction data and literature values for the single crystal resistivity tensor are of the same order of magnitude as the experimentally-measured resistivities along the longitudinal, radial, and tangential directions of the Bi-2212 tube. The small resistive anisotropy ratio in MCP Bi-2212 samples is the consequence of weak textures, with maxima of orientation distributions not larger than a few m.r.d. However, the very large single crystal anisotropy partly compensates the low texture strengths, and additional simulations confirm that significant effects on the conducting properties can be expected—even with low orientation degrees, when strong anisotropy is intrinsically present.

Though the resistivity data are not directly relevant to superconducting properties, the relationship between the measured and calculated resistivities opposite to anticipation and the temperature dependence of normalized resistivity that coincides with that of in-plane resistivity of single crystals point out the percolative nature of current transport confined mostly to the *ab*-planes, which is likely due to special nature of high-angle grain boundaries in this material. The existence of such boundaries could be the key for understanding high J_c_ values in the MCP bulk Bi-2212.

## Supplementary Materials

Supplementary File 1

## Appendix A

The main equations for the calculation of the geometric average of the resistivity second-rank tensor over crystallite orientations are given here. The macroscopic resistivity tensor of a textured polycrystal with an orientation distribution given by f(g)—where g is a set of three Euler angles relating crystal and sample reference frames [31]—is obtained from (<…>: arithmetic mean; […]: geometric mean; …^M^: macroscopic value):

ρ_ij_^M^ = [ρ]_ij_ = exp(<lnρ>_i’j’_) = exp(<Θ>_ij_,_i’j’_ (lnρ)_i’j’_), (A1)

with

(A2) $${<\Theta >}_{\mathrm{ij},i^{\prime }j^{\prime }}=\underset{g}{\int }\Theta_{i}^{i^{\prime }}\left(g\right)\Theta_{j}^{j^{\prime }}\left(g\right)\;f\left(g\right)\mathrm{dg}$$

and

(A3) $${\left(\ln\rho \right)}_{i^{\prime }j^{\prime }}=\overset{3}{\underset{\lambda =1}{\sum }}\ln\left(\rho^{\left(\lambda \right)}\right){b_{i}}^{\left(\lambda \right)}{b_{j}}^{\left(\lambda \right)}$$

(A4) $${\left(\ln\rho \right)}_{i^{\prime }j^{\prime }}=\ln\overset{3}{\underset{\lambda =1}{\prod }}{\left(\rho^{\left(\lambda \right)}\right)}^{{b_{i}}^{\left(\lambda \right)}{b_{j}}^{\left(\lambda \right)}}$$

where the (λ) exponents denote eigenvalues of the corresponding quantities, and the b_i_^(λ)^, b_j_^(λ)^ are the eigentensors resulting from diagonalisation of the arithmetic average <lnρ>_i’j’_ for the oriented polycrystal [32]. The two successive tensor transformations Θ_i_^i’^ relate to the 2^nd^ order resistivity tensor character. The factorial entering the calculation explains the term “geometric mean”, in the sense that the oriented polycrystal macroscopic resistivity is obtained by the mean averaging of the single crystal resistivity eigenvalues. Similar expressions could be obtained for the macroscopic conductivity tensor σ_ij_^M^, which admits as eigenvalues σ^(λ)^ = 1/ρ^(λ)^ the reciprocal of the resistivity eigenvalues. This warrants that the same macroscopic electrical properties are calculated either using resistivity or conductivity in the geometric mean average approach. In other words, invertibility is ensured: the average of the inverse macroscopic property is consistent with the inverse of the average macroscopic property.
