# Supplementary material for: Preferred Orientation Contribution to the Anisotropic Normal State Resistivity in Superconducting Melt-Cast Processed Bi2Sr2CaCu2O8+δ
Source: Materials (Basel). 2017 May 15;10(5):534. doi: 10.3390/ma10050534 (PMC5459035; doi:10.3390/ma10050534)
Supplement: Supplementary File 1 [file materials-10-00534-s001.pdf]

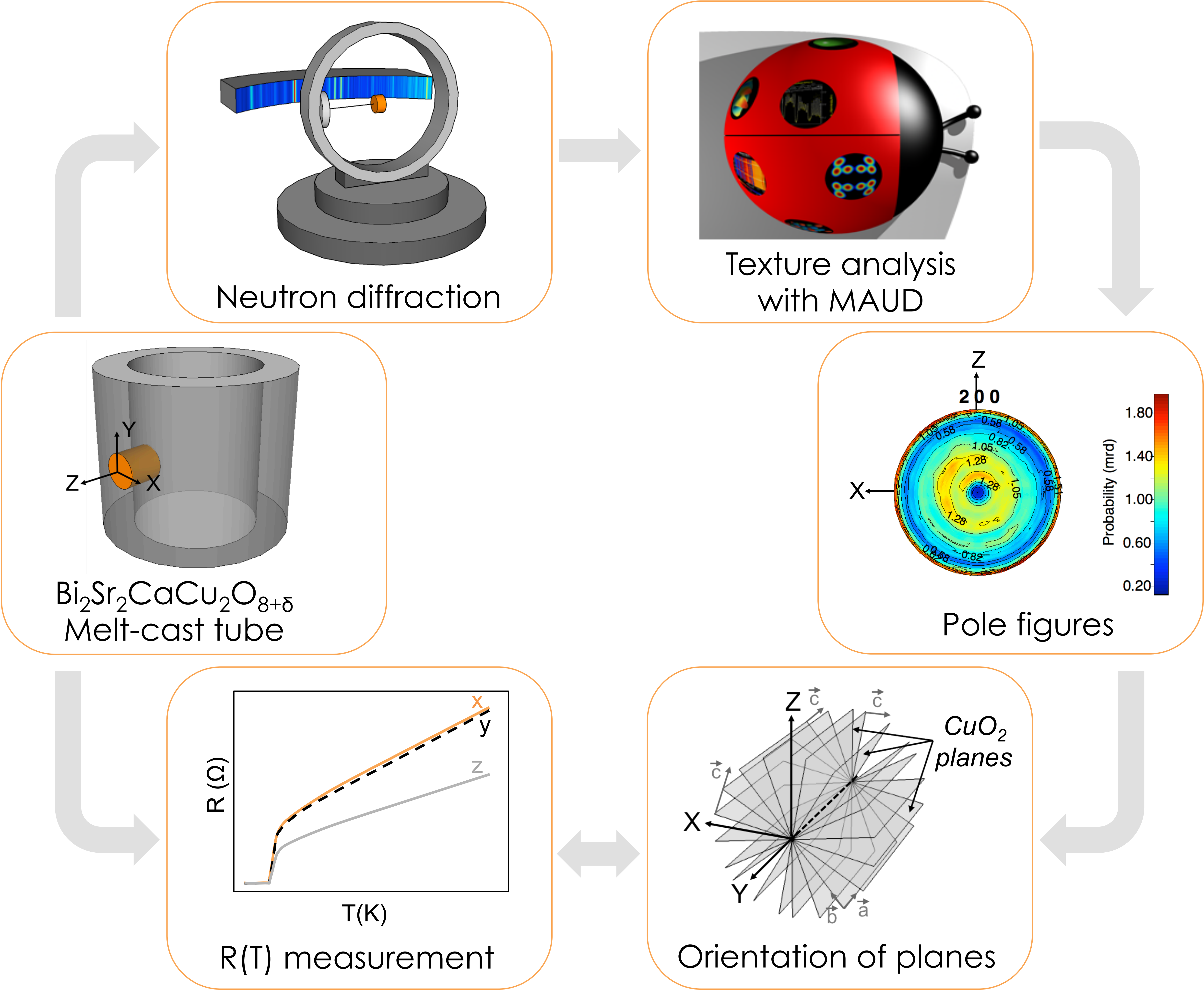
Neutron diffraction
Texture analysis with MAUD
Pole figures
Z
2 0 0
X
Probability (mrd)
1.80
1.40
1.00
0.60
0.20
Orientation of planes
Z
X
Y
c
b
a
CuO2 planes
R(T) measurement
R (Ω)
T(K)
x
y
z
Bi2Sr2CaCu2O8+δ
Melt-cast tube
Y
Z
X
